# α-Ketoglutarate Attenuates Oxidative Stress-Induced Neuronal Aging via Modulation of the mTOR Pathway

**DOI:** 10.3390/ph18081080

**Published:** 2025-07-22

**Authors:** Ruoqing Guan, Zhaoyun Xue, Kaikun Huang, Yanqing Zhao, Gongyun He, Yuxing Dai, Mo Liang, Yanzi Wen, Xueshi Ye, Peiqing Liu, Jianwen Chen

**Affiliations:** 1 National and Local United Engineering Lab of Druggability and New Drugs Evaluation, School of Pharmaceutical Sciences, Sun Yat-sen University, Guangzhou 510006, China; guanrq@mail2.sysu.edu.cn (R.G.); xuezhy3@mail.sysu.edu.cn (Z.X.); hegy26@mail2.sysu.edu.cn (G.H.); daiyx5@mail2.sysu.edu.cn (Y.D.); liangm55@mail.sysu.edu.cn (M.L.); wenyz@mail.sysu.edu.cn (Y.W.); yexsh6@mail.sysu.edu.cn (X.Y.); 2 Guangdong Province Engineering Laboratory for Druggability and New Drug Evaluation, School of Pharmaceutical Sciences, Sun Yat-sen University, Guangzhou 510006, China; 3 Shenzhen Xintianhe Biotechnology Co., Ltd., Shenzhen 518057, China; huangkaikun@kexing.com (K.H.); zhaoyanqing@kexing.com (Y.Z.); 4 Shenzhen Xintianhe Biotechnology Co., Ltd., Jinan 250215, China; 5 Sun Yat sen University Nanchang Research Institute, Nanchang 330096, China

**Keywords:** alpha-ketoglutarate, brain aging, H_2_O_2_, D-galactose, proteomic, TCA, mTOR

## Abstract

**Background/Objectives**: Oxidative stress constitutes a principal pathophysiological mechanism driving neurodegeneration and brain aging. α-Ketoglutarate (AKG), a key intermediate of the tricarboxylic acid (TCA) cycle, has shown potential in longevity and oxidative stress resistance. However, the role of AKG in oxidative stress-induced neuronal senescence and its interaction with the mTOR signaling pathway during neuronal aging remain poorly understood, posing a key challenge for developing senescence-targeted therapies. **Methods**: We investigated the neuroprotective effects of AKG using H_2_O_2_-induced senescence in HT22 cells and a D-galactose-induced brain aging mouse model. Assessments encompassed SA-β-gal staining, EdU incorporation, mitochondrial membrane potential (JC-1), and ROS measurement. Antioxidant markers, ATP levels, and the NAD^+^/NADH ratio were also analyzed. Proteomic profiling (DIA-MS) and KEGG/GSEA enrichment analyses were employed to identify AKG-responsive signaling pathways, and Western blotting validated changes in mTOR signaling and downstream effectors. **Results**: AKG significantly alleviated H_2_O_2_-induced senescence in HT22 cells, evidenced by enhanced cell viability, reduced ROS level, restored mitochondrial function, and downregulated p53/p21 expression. In vivo, AKG administration improved cognitive deficits and vestibulomotor dysfunction while ameliorating brain oxidative damage in aging mice. Proteomics revealed mTOR signaling pathways as key targets for AKG’s anti-aging activity. Mechanistically, AKG suppressed mTOR phosphorylation and activated ULK1, suggesting modulation of autophagy and metabolic homeostasis. These effects were accompanied by enhanced antioxidant enzyme activities and improved redox homeostasis. **Conclusions**: Our study demonstrates that AKG mitigates oxidative stress-induced neuronal senescence through suppression of the mTOR pathway and enhancement of mitochondrial and antioxidant function. These findings highlight AKG as a metabolic intervention candidate for age-related neurodegenerative diseases.

## 1. Introduction

Aging emerges as a principal etiological determinant in neurodegenerative diseases [1,2], including Alzheimer’s disease, Parkinson’s disease, and aging-associated cognitive decline [3,4]. These disorders are pathologically characterized by progressive neuronal depletion and declining brain functionality, imposing substantial socioeconomic burdens [5,6,7]. Mechanistically, oxidative stress has been identified as a pivotal regulatory nexus governing both neuronal aging and degeneration [8,9]. This pathological pathway disrupts redox homeostasis through excessive generation of reactive oxygen species (ROS) [10], thereby compromising cellular structural integrity and ultimately activating aging-associated pathways [11]. Consequently, the development of targeted therapeutic strategies to mitigate oxidative stress represents a critical imperative for addressing age-related neurodegenerative disorders.

α-Ketoglutarate (AKG), a pivotal tricarboxylic acid (TCA) cycle intermediate [12], has emerged as a multifunctional metabolite with pleiotropic roles in cellular metabolism [13], redox homeostasis, and aging regulation [13,14]. Functioning as both an endogenous metabolic hub and signaling molecule, AKG orchestrates nitrogen flux in amino acid metabolism, serves as a co-substrate for epigenetic regulators, and modulates mitochondrial function [15]. Recent studies have demonstrated that AKG possesses pleiotropic biological effects, including promotion of longevity [16], attenuation of inflammation [17], and enhancement of oxidative stress resilience [18]. These effects are thought to be mediated through its regulatory roles in mitochondrial metabolism, antioxidant defense, and immune modulation. However, while these promising attributes are well-documented, the mechanisms underlying AKG’s neuroprotective effects—especially under oxidative stress—remain incompletely mapped, representing a knowledge gap in translating metabolic interventions to neurodegenerative therapeutics.

The mTOR pathway is a key regulatory network that maintains cellular homeostasis by integrating multiple signals involved in cell growth, metabolism, and autophagy [19,20]. Sustained activation of mTOR signaling has been shown to be closely associated with senescence and neurodegenerative disorders, whereas its inhibition can attenuate aging and enhance neurological function [21,22]. Metabolically, mTOR is widely recognized as a potential target for interventions against oxidative stress-related aging [23,24]. In this study, we found that AKG exerted significant neuroprotective effects in brain aging via modulating the mTOR signaling pathway. This finding provides new insights and potential strategies for targeted regulation of the mTOR pathway.

## 2. Results

### 2.1. Reversal of D-Gal-Induced Brain Aging in C57BL/6 Mice by AKG

**Figure 1:**
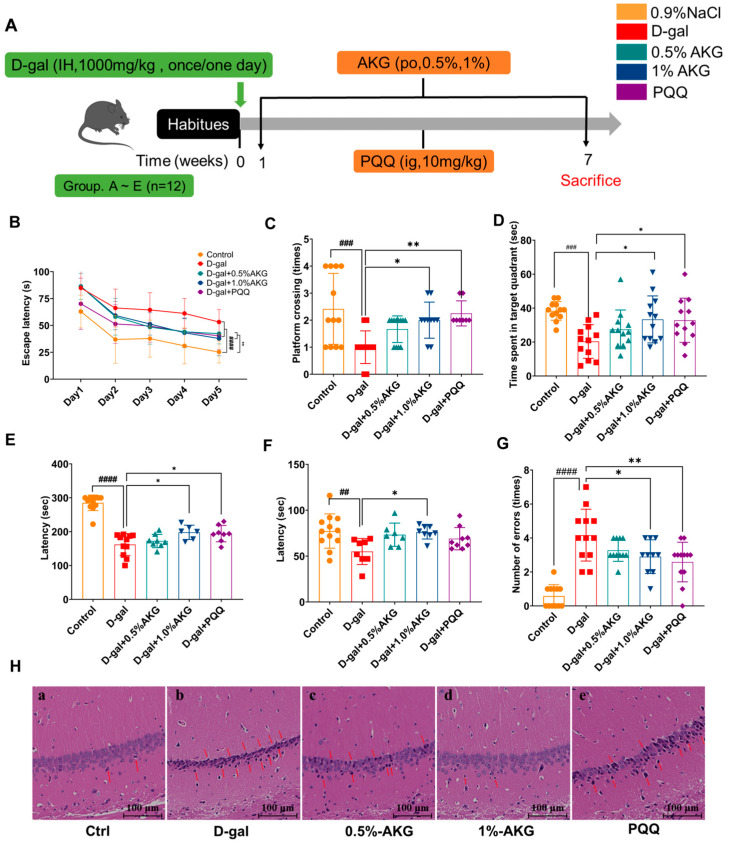
AKG improves behavior and alleviates brain damage in D-gal-induced aging mice. (**A**) Schematic diagram of the animal study design. (**B**) The mean escape latency (5 days) to find the hidden platform in place navigation test. (**C**) Number of platform crossings. (**D**) Time spent in target quadrant. (**E**) Enhancement in remaining time in rotarod test. (**F**,**G**) Latency and number of errors in passive avoidance test. (**H**) Effect of AKG treatment on brain histopathological alterations with H&E staining: (**a**) control group; (**b**) model group; (**c**) 0.5% AKG group; (**d**) 1% AKG group; (**e**) PQQ group. All data are expressed as means ± S.D. (*n* = 6). ^##^ *p* < 0.01, ^###^ *p* < 0.001, and ^####^ *p* < 0.0001 versus the control group; * *p* < 0.05 and ** *p* < 0.01 versus the model group.

The neuroprotective efficacy of AKG treatment against D-gal-induced brain aging in C57BL/6 mice was systematically evaluated through behavioral phenotyping and neurohistological evaluation (experimental design: Figure 1A). Morris water maze analyses revealed that AKG dose-dependently reduced mean escape latency during the 5-day place navigation phase (Figure 1B) while enhancing platform crossing frequency during probe trials (Figure 1C), indicating improved spatial cognitive performance. Moreover, AKG treatment exhibited significantly prolonged target quadrant dwell time (Figure 1D), further confirming enhanced memory consolidation. Rotarod testing demonstrated extended latency to fall after AKG treatment (Figure 1E), indicating preservation of vestibulomotor function and balance. Passive avoidance tests (Figure 1F,G) showed elevated shock zone entry latency and reduced error frequency in AKG groups (Figure 1F,G). Histopathological quantification of H&E-stained brain tissue (Figure 1H) revealed significant neurodegeneration in AKG-treated mice, with a maximal neuroprotection effect observed at 1% supplementation.

### 2.2. AKG Attenuates Brain and Plasma Oxidative Stress in D-Gal-Induced Aging Mice

**Figure 2:**
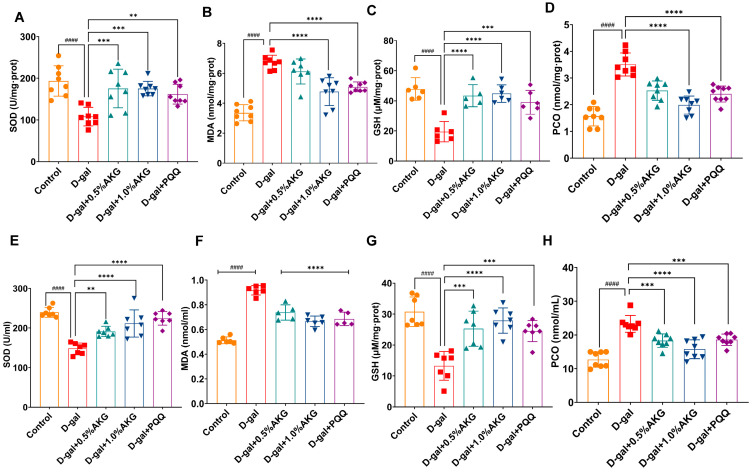
AKG modulates oxidative stress markers in the brain and plasma of D-gal-induced aging mice. (**A**–**D**) Levels of superoxide dismutase (SOD), malondialdehyde (MDA), glutathione (GSH), and protein carbonyl (PCO) in the brain. (**E**–**H**) Levels of SOD, MDA, GSH, and PCO in the plasma. All data are expressed as means ± S.D. (*n* = 6). ^####^ *p* < 0.0001 versus the control group; ** *p* < 0.01, *** *p* < 0.001 and **** *p* < 0.0001 versus the model group.

AKG treatment significantly dose-dependently alleviated oxidative stress in both the brain and plasma of D-gal-induced aging mice. In brain tissue, AKG-enhanced antioxidant was observed through elevated superoxide dismutase (SOD) and glutathione (GSH) levels in a dose-dependent manner (Figure 2A,C), concurrent with reduced lipid peroxidation (malondialdehyde, MDA; Figure 2B) and protein oxidation (protein carbonyls, PCO; Figure 2D), indicating enhanced antioxidant defenses and reduced lipid and protein oxidative damage. Plasma profiling recapitulated these redox-modulatory effects, showing increased SOD/GSH (Figure 2E,G) and decreased MDA/PCO levels (Figure 2F,H) after AKG administration. These complementary findings establish AKG’s dual capacity to preserve systemic oxidative homeostasis while mitigating central nervous system redox imbalance in a dose-dependent manner.

### 2.3. AKG Improves Mitochondrial Function in D-Gal-Induced Aging Mice

**Figure 3:**
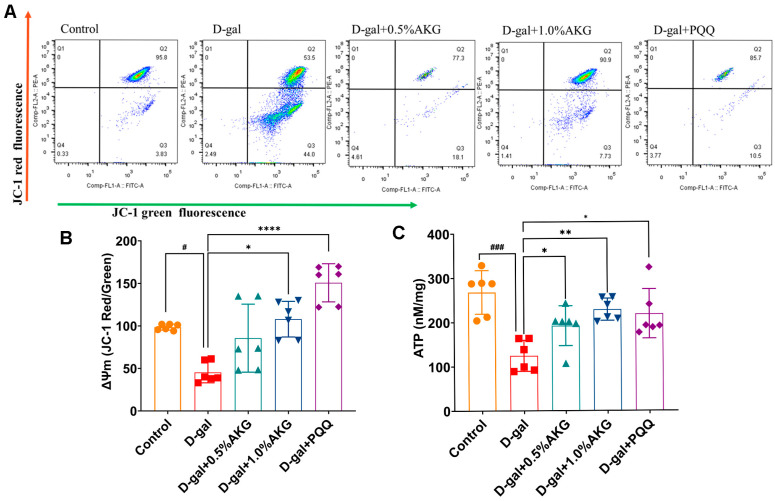
AKG restores mitochondrial activity and integrity in the brain of aging mice. (**A**) Mitochondrial membrane potential (MMP) assessed by JC-1 fluorescence staining. (**B**) Quantitative analysis of the red-to-green fluorescence ratio following JC-1 staining. (**C**) ATP levels in each group. All data are expressed as means ± S.D. (*n* = 6). ^#^ *p* < 0.05 and ^###^ *p* < 0.001 versus the control group; * *p* < 0.05, ** *p* < 0.01 and **** *p* < 0.0001 versus the model group.

AKG treatment markedly ameliorated mitochondrial dysfunction in the brain of D-gal-induced aging mice. JC-1 staining showed that AKG increased mitochondrial membrane potential (MMP), as indicated by a higher red-to-green fluorescence ratio (Figure 3A,B), indicating restored mitochondrial transmembrane potential homeostasis. Concomitantly, AKG dose-dependently normalized cerebral ATP production to physiological levels (Figure 3C), confirming bioenergetic competence recovery. These findings collectively establish AKG’s capacity to counteract senescence-associated mitochondrial deterioration through modulation of structural integrity.

### 2.4. AKG Protects HT22 Cells Against H_2_O_2_-Induced Senescence and Promotes Cell Viability

**Figure 4:**
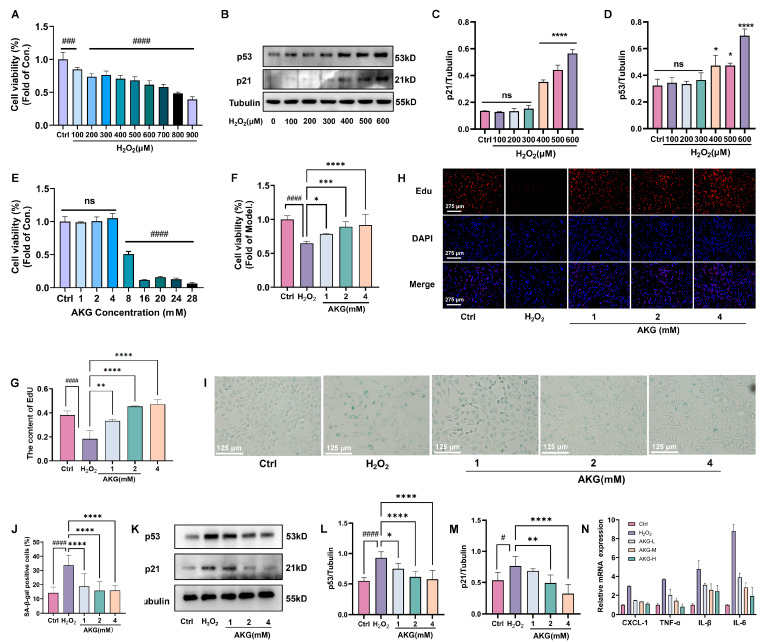
AKG attenuates H_2_O_2_-induced senescence in HT22 cells via modulation of cell viability, senescence markers, and the p53/p21 pathway. (**A**) H_2_O_2_ treatment significantly reduced HT22 cell viability in a dose-dependent manner. (**B**) Western blot analysis of p53 and p21 expression following H_2_O_2_ treatment. (**C**,**D**) Quantitative analysis of p53 and p21 protein expression levels. (**E**) Effects of different concentrations of AKG on cell viability in HT22 cells. (**F**) Effects of different concentrations of AKG on cell viability in H_2_O_2_-induced HT22 cells. (**G**,**H**) EdU staining and quantification of proliferating HT22 cells. (**I**,**J**) SA-β-gal staining and quantification. (**K**) The H_2_O_2_-induced aging, reversed by AKG, was detected by western blot (p53 and p21). (**L**,**M**) Quantification of p53 and p21 protein expression levels. (**N**) The mRNA expression levels of CXCL-1, TNF-α, IL-1β, and IL-6 were quantified by qRT-PCR. All data are expressed as means ± S.D. (*n* ≥ 3). ^#^ *p* < 0.05, ^###^ *p* < 0.001, and ^####^ *p* < 0.0001 versus the control group; * *p* < 0.05, ** *p* < 0.01, *** *p* < 0.001, and **** *p* < 0.0001 versus the model group.

H_2_O_2_ treatment significantly reduced HT22 cell viability in a dose-dependent manner (Figure 4A), concomitant with upregulated expression of senescence-associated proteins p53 and p21 (Figure 4B–D). AKG monotherapy exhibited no adverse effects on cellular viability while enhancing basal proliferative capacity at the tested concentration (Figure 4E). Furthermore, AKG pretreatment significantly dose-dependently reversed H_2_O_2_-induced cytotoxicity (Figure 4F). EdU staining revealed AKG-mediated restoration of replicative competence in senescent HT22 cells (Figure 4G,H), paralleled by marked attenuation in the number of senescent cells following AKG treatment (Figure 4I,J). Western blot analysis further confirmed that AKG downregulated H_2_O_2_-induced overexpression of p53 and p21 (Figure 4K–M). Additionally, AKG significantly suppressed the senescence-associated secretory phenotype (SASP), as demonstrated by reduced secretion of pro-inflammatory mediators (CXCL-1, TNF-α, IL-1β, and IL-6) in HT22 (*p* < 0.05, Figure 4N).

### 2.5. AKG Alleviates Oxidative Stress and Promotes Mitochondrial Metabolism in H_2_O_2_-Induced HT22 Cells

**Figure 5:**
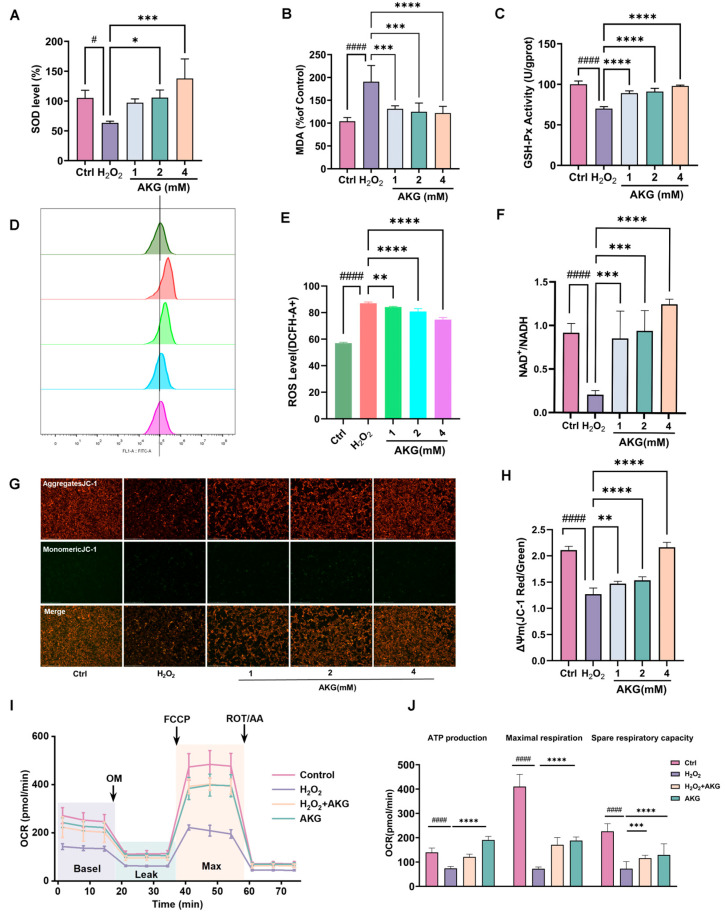
AKG modulates oxidative stress, mitochondrial function, and NAD+ metabolism in H_2_O_2_-induced HT22 Cells. (**A**) Effects of AKG on SOD, (**B**) MDA, and (**C**) GSH levels in H_2_O_2_-induced HT22 cells. (**D**,**E**) Flow cytometry to detect the effect of AKG on ROS levels. (**F**) AKG treatment increased the NAD^+^/NADH ratio in HT22 cells. (**G**,**H**) JC-1 staining and quantification of mitochondrial membrane potential (MMP). Magnification: 10×, scale bar = 275 μm. (**I**) OCR changes during mitochondrial respiration. (**J**) Quantitation of ATP-production coupled respiration, maximal respiration, and spare respiratory capacity *n* = 6. All data are expressed as means ± S.D. (*n* ≥ 3). ^#^ *p* < 0.05 and ^####^ *p* < 0.0001 versus the control group; * *p* < 0.05, ** *p* < 0.01, *** *p* < 0.001, and **** *p* < 0.0001 versus the model group.

AKG treatment significantly enhanced SOD (Figure 5A) and GSH activity (Figure 5C), while suppressing MDA accumulation (Figure 5B) in H_2_O_2_-induced HT22 cells, demonstrating its capacity to ameliorate oxidative damage. Flow cytometry analysis showed a marked decrease in intracellular ROS levels following AKG treatment (Figure 5D,E). Concurrently, AKG treatment elevated the NAD^+^/NADH ratio (Figure 5F), indicative of restored redox homeostasis and mitochondrial metabolic competence. JC-1 staining analysis demonstrated that AKG restored mitochondrial membrane potential, as evidenced by increased red/green fluorescence ratios (Figure 5G,H). Mitochondrial respiration, as reflected by the oxygen consumption rate (OCR), serves as a critical biomarker of cellular energy homeostasis. Using a mitochondrial stress assay, we observed that H_2_O_2_-exposed HT22 cells exhibited a marked suppression (*p* < 0.01) in baseline OCR and ATP-linked respiration compared to controls. Strikingly, AKG administration significantly attenuated these deficits (*p* < 0.05, Figure 5I,J), suggesting a restorative effect on mitochondrial bioenergetics under oxidative challenge. These findings collectively delineate AKG’s multi-modal cytoprotective mechanism involving oxidative stress mitigation and mitochondrial functional reinforcement.

### 2.6. AKG Regulates the mTOR and p53 Pathways, as Revealed by Proteomics and Verified by Western Blot

**Figure 6:**
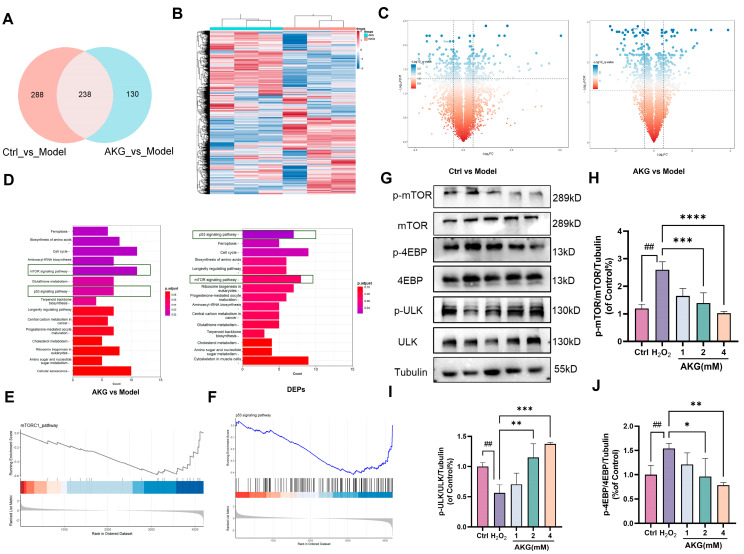
Proteomics analysis and validation reveal that AKG modulates mTOR and p53 signaling pathways. (**A**) Venn diagram showing the overlap of differentially expressed proteins (DEPs) between Ctrl vs. model and AKG vs. model comparisons. (**B**) Heatmap of DEPs in AKG-treated versus H_2_O_2_-induced HT22 cells. (**C**) Volcano plots showing significantly altered proteins (|log_2_FC| > 1, * *p* < 0.05) in Ctrl vs. model and AKG vs. model groups. (**D**) KEGG pathway enrichment analysis of DEPs in AKG vs. model (left) and the intersecting 238 proteins (right), highlighting pathways including mTOR and p53 signaling. (**E**,**F**) Gene set enrichment analysis (GSEA) indicated a negative enrichment of mTOR and p53 signaling pathways following AKG treatment. (**G**) Representative Western blot images of mTOR signaling-related proteins in HT22 cells. (**H**–**J**) Quantitative analysis of p-mTOR/mTOR, p-ULK1/ULK1, and p-4EBP/4EBP levels normalized to Tubulin. All data are expressed as means ± S.D. (*n* ≥ 3). ^##^ *p* < 0.01 versus the control group; * *p* < 0.05, ** *p* < 0.01, *** *p* < 0.001, and **** *p* < 0.0001 versus the model group.

To further elucidate AKG’s molecular mechanisms in counteracting oxidative stress-driven neuronal senescence, we performed a proteomic analysis in H_2_O_2_-induced HT22 cells. Differential expression analysis identified 238 overlapping protein targets across comparative group contrasts (Ctrl vs. model; AKG vs. model) (Figure 6A). Heatmap visualization revealed AKG-specific differential proteomic signatures distinct from pathological profiles (Figure 6B). Volcano plots confirmed statistically robust differential expression (|log_2_FC| > 1, * *p* < 0.05) across experimental paradigms (Figure 6C), highlighting AKG’s functional convergence on senescence regulatory networks.

KEGG pathway enrichment analysis revealed that AKG exerted pathway-specific modulation across core senescence-associated signaling—especially mTOR/p53 signaling, glutathione metabolism, and ferroptosis regulation (Figure 6D, left). Notably, the 238 intersecting proteins were also enriched in mTOR and p53 signaling pathways (Figure 6D, right). Consistently, gene set enrichment analysis (GSEA) revealed a downregulation trend in mTOR and p53 pathways following AKG treatment (Figure 6E,F).

To validate the proteomic findings, we performed Western blot analysis of key proteins involved in the mTOR signaling pathway. AKG treatment dose-dependently decreased the phosphorylation levels of mTOR (Ser2448) and its canonical substrate 4EBP (Thr37/46) (Figure 6J), suggesting inhibition of mTOR pathway activity. In addition, ULK1 phosphorylation exhibited a progressive increase (Figure 6I), potentially linked to mTOR suppression, though its functional role in AKG-mediated anti-senescence requires further mechanistic validation. These results collectively indicate that AKG attenuates oxidative stress-induced cellular senescence through coordinated modulation of mTOR and p53 signaling pathways.

## 3. Discussion

Aging is a major etiological factor in various neurodegenerative diseases, primarily linked to excessive reactive oxygen species (ROS) accumulation, mitochondrial dysfunction, and chronic inflammation [25,26,27]. α-Ketoglutarate (AKG), a key endogenous intermediate in the tricarboxylic acid (TCA) cycle, has gained attention as a multifunctional metabolite with strong anti-inflammatory, antioxidant, and geroprotective effects [28]. In this study, we developed an H_2_O_2_-induced oxidative stress model to systematically evaluate AKG’s anti-senescence properties in neuronal models [29,30]. Mechanistic studies revealed that AKG attenuates oxidative stress-induced cellular aging through mTOR pathway suppression, elucidating its dual function as both a metabolic regulator and geroprotective agent. These findings establish a novel mechanistic foundation for AKG-mediated neuroprotection, highlighting its potential application in developing interventions against age-associated neurodegenerative diseases.

Oxidative stress is mechanistically implicated as a principal etiological driver in neurodegenerative pathogenesis, with pathological ROS accumulation directly contributing to redox homeostasis perturbation, cellular oxidative damage, and irreversible activation of pro-senescence signaling [31,32,33]. Our mechanistic investigation revealed that AKG pharmacologically attenuates H_2_O_2_-induced pathological ROS generation while concomitantly restoring cellular function. In parallel, AKG also exerted anti-inflammatory effects, as evidenced by the downregulation of senescence-associated secretory phenotype (SASP) factors. This suggests that AKG may mitigate not only oxidative injury but also chronic inflammation—two interlinked drivers of neurodegeneration. This dual functionality—as both a redox buffer and metabolic regulator—positions AKG as a novel therapeutic antioxidant for combating oxidative stress-associated neurodegeneration through simultaneous antioxidant activity, inflammation resolution, and enhancement of cellular resilience.

The mTOR signaling pathway operates as a master regulatory hub governing cellular growth, metabolism, and autophagy, with its sustained upregulation being pathologically implicated in accelerated aging phenotypes and neurodegenerative disorders [34,35,36]. In our experiments, pharmacological AKG administration attenuates mTOR signaling activation, evidenced by dose-dependent reductions in mTORC1 complex phosphorylation and diminished activation of its downstream target 4EBP1. This mTOR-driven aging suppression mechanistically validates AKG’s ability to counteract oxidative stress-induced neuronal aging.

Oxidative stress is known to activate mTOR signaling through suppression of AMPK and activation of the PI3K/Akt pathway, thereby impairing autophagy and accelerating cellular senescence [37,38]. Our findings support this model, showing that H_2_O_2_-induced neuronal senescence is accompanied by mTOR activation and is reversed by AKG treatment. Notably, given the reciprocal relationship between oxidative stress and mTOR activity, we propose that mTOR inhibition may occur downstream of AKG’s antioxidant effects, serving as a key regulatory node linking redox homeostasis and autophagy activation. Similar AKG-mediated modulation of oxidative stress and mTOR signaling has been reported in other pathological models. For example, a study in Drosophila demonstrated that AKG extends lifespan by directly inhibiting mTOR and activating AMPK [39], suggesting that redox- and nutrient-sensing pathways may both contribute to AKG’s effects depending on context.

Overall, our findings demonstrate that AKG attenuates oxidative stress-induced neuronal senescence through mTOR pathway suppression, with potential involvement of autophagy-related signaling in enhancing neuronal resilience. This study highlights AKG as a metabolic regulator that engages the redox–mTOR axis, offering mechanistic insight into its senescence-targeting potential. While our data support a model in which mTOR modulation is secondary to redox improvement, the bidirectional interplay between oxidative stress, mitochondrial function, and nutrient-sensing pathways warrants further exploration. Clarifying the temporal and cell-type-specific dynamics of AKG action will be critical for translating its neuroprotective potential into therapeutic applications for age-related neurodegenerative diseases.

Future studies should validate AKG’s therapeutic effects in prototypical neurodegenerative models, particularly Alzheimer’s and Parkinson’s diseases. Complementary investigations must elucidate AKG’s regulatory mechanisms on autophagy, mitochondrial bioenergetics, and metabolic pathway interactions to comprehensively decipher its neuroprotective orchestration.

## 4. Materials and Methods

### 4.1. Cell Culture and Treatments

HT22 cells (mouse hippocampal neuronal cell line) were purchased from Procell Life Science & Technology Co., Ltd. (Wuhan, China; Cat# CL-0410). Cells were cultured in high-glucose Dulbecco’s Modified Eagle Medium (DMEM; Gibco (Grand Island, NY, USA), Cat# 11965092) supplemented with 10% fetal bovine serum (FBS; Vazyme (Nanjing, Jiangsu, China), Cat# 900-00-10, Nanjing, China), 100 U/mL penicillin, and 100 µg/mL streptomycin, and maintained at 37 °C in a humidified incubator with 5% CO_2_. Cells were passaged at 80–90% confluence using 0.25% trypsin-EDTA solution (NCM Biotech (Suzhou, Jiangsu, China), Cat# C0201, Suzhou, China). To induce oxidative stress, cells were treated with 400 µM hydrogen peroxide (H_2_O_2_; Sigma-Aldrich (St. Louis, MO, USA), Cat# 323381) for 24 h. The experimental design included a control group, a model group, and several α-ketoglutarate (Xintianhe, Shenzhen, China) treatment groups. The control group received only complete medium with 10% FBS. The model group was exposed to 400 µM H_2_O_2_ in complete medium. The AKG treatment groups were co-treated with 400 µM H_2_O_2_ and varying concentrations of AKG (1, 2, and 4 mM) for 24 h.

### 4.2. Measurement of Antioxidant Enzyme Activities

SOD activity in brain tissue was determined using a WST-8-based assay kit (Beyotime, Cat# S0101, Shanghai, China) according to the manufacturer’s instructions. Absorbance was measured at 450 nm after 30 min incubation at 37 °C. GSH-Px activity was assessed using a NADPH-coupled kit (Beyotime, Cat# S0058, Shanghai, China). GSH-Px catalyzes the reduction of H_2_O_2_, consuming GSH and oxidizing NADPH, which decreases absorbance at 340 nm.

### 4.3. Malondialdehyde (MDA) Analysis

Malondialdehyde (MDA) levels were measured using a commercial assay kit (Beyotime, Cat# S0131S, Shanghai, China) based on the thiobarbituric acid (TBA) method. Briefly, MDA reacts with TBA to form a red-colored MDA-TBA adduct, which is quantified by colorimetric analysis. The absorbance was recorded at 532 nm using a microplate reader.

### 4.4. Determination of Protein Carbonyl Levels

Protein carbonyl (PCO) levels in brain tissue were measured using a colorimetric assay kit (Elabscience, Cat# E-BC-K730-M, Wuhan, China) according to the manufacturer’s instructions. This method is based on the reaction between carbonyl groups and 2,4-dinitrophenylhydrazine (DNPH) to form hydrazones, which generate a measurable absorbance at 370 nm. Tissue homogenates were prepared and treated with DNPH or control reagents, followed by a series of washing and denaturation steps to remove interfering substances. After solubilization of the final precipitate, absorbance was measured at 370 nm using a microplate reader. Protein concentrations were determined using a BCA Protein Assay Kit (Beyotime, Cat# P0012, Shanghai, China) for normalization.

### 4.5. Cell Viability Assay (CCK-8)

Cell viability was determined using the Cell Counting Kit-8 (Beyotime, Cat# C0038, Shanghai, China) following the manufacturer’s instructions. HT22 cells were seeded into 96-well plates at an appropriate density and incubated for 24 h. After treatment, 10 μL of CCK-8 reagent was added to each well, followed by incubation at 37 °C for 1 h. The absorbance at 450 nm was measured using a microplate reader. Cell viability was calculated based on optical density (OD) values and normalized to the control group.

### 4.6. Intracellular ROS Detection

Intracellular reactive oxygen species (ROS) levels were measured using a DCFH-DA-based ROS assay kit (Beyotime, Cat# S0033S, Shanghai, China) according to the manufacturer’s instructions. DCFH-DA is a non-fluorescent probe that is converted into fluorescent DCF upon oxidation by ROS, allowing quantitative detection of intracellular ROS.

HT22 cells were collected after treatment, washed twice with PBS, and incubated with 10 μM DCFH-DA at 37 °C for 20 min in the dark. After incubation, cells were washed thoroughly with PBS to remove excess dye. The fluorescence intensity of DCF was analyzed using a flow cytometer (FITC channel, excitation: 488 nm, emission: 525 nm). Mean fluorescence intensity (MFI) was used to quantify ROS levels.

### 4.7. Mitochondrial Membrane Potential (Δψm) Assay

Mitochondrial membrane potential (Δψm) was assessed using a JC-1 staining kit (Elabscience, Cat# E-CK-A201, Wuhan, China) according to the manufacturer’s instructions. JC-1 is a cationic dye that accumulates in mitochondria in a membrane potential-dependent manner. In healthy cells with high Δψm, JC-1 aggregates emit red fluorescence, while in depolarized mitochondria with low Δψm, JC-1 remains in monomeric form and emits green fluorescence. The red-to-green fluorescence ratio reflects changes in Δψm.

After treatment, HT22 cells were washed with PBS and incubated with JC-1 working solution at 37 °C for 20 min in the dark. After staining, cells were washed and imaged immediately using an integrated fluorescence imaging system. Red fluorescence (aggregates) and green fluorescence (monomers) were detected using appropriate filter sets. The Δψm was quantified by calculating the ratio of red to green fluorescence intensity using ImageJ software (NIH, Santa Clara, CA, USA; Version number: 1.54f).

### 4.8. Measurement of Oxygen Consumption Rate (OCR)

The oxygen consumption rate (OCR) of HT22 cells was measured using the Seahorse XFe96 Extracellular Flux Analyzer (Agilent Technologies, Santa Clara, CA, USA) to evaluate mitochondrial respiratory function. Cells were seeded into XFe96-well microplates (Agilent, Cat. No. 101104-100) at a density of 8 × 10^4^ cells/well and cultured overnight to ensure adhesion. Before the assay, the culture medium was replaced with Seahorse XF Base Medium (Agilent, Cat. No. 103334-100), supplemented with 2 mM glutamine, 10 mM glucose, and 1 mM sodium pyruvate (all from Agilent), and adjusted to pH 7.4. The cells were incubated in a non-CO_2_ incubator at 37 °C for 1 h to allow temperature and pH equilibration.

Mitochondrial respiration was assessed using the Seahorse XF Cell Mito Stress Test Kit (Agilent, Cat. No. 103015-100), according to the manufacturer’s instructions. OCR was measured in real time after the sequential injection of oligomycin (1 μM), FCCP (1 μM), and a combination of rotenone (0.5 μM) and antimycin A (0.5 μM), all included in the kit. Key parameters—including basal respiration, ATP production, maximal respiration, spare respiratory capacity, and proton leak—were calculated using Wave software (Agilent Technologies; Version number: 2.6). All experiments were conducted in at least six independent replicates.

### 4.9. Senescence-Associated β-Galactosidase (SA-β-Gal) Staining

Cellular senescence was evaluated using a senescence-associated β-galactosidase (SA-β-Gal) staining kit (Beyotime, Cat# C0602, Shanghai, China) according to the manufacturer’s instructions. HT22 cells were seeded in 6-well plates and treated as indicated. After treatment, cells were washed with PBS, fixed with the provided fixative solution for 15 min at room temperature, and then incubated with SA-β-Gal staining solution at 37 °C overnight in a dry incubator (no CO_2_). The following day, blue-stained senescent cells were observed under an inverted microscope and counted in randomly selected fields. The percentage of SA-β-Gal-positive cells was calculated from three independent experiments.

### 4.10. Western Blotting

HT22 cells’ total protein was extracted using RIPA buffer (Beyotime, Cat# P0013B, Shanghai, China), separated on 10% SDS-PAGE, and transferred to PVDF membranes (Millipore, Cat# IPVH00010, Burlington, MA, USA). These membranes were then blocked with 5% skimmed milk, washed with TBST (Tris-Buffered Saline with Tween^®^ 20, Beyotime, Shanghai, China), and incubated overnight at 4 °C with primary antibodies, including p53 (1:1000), p21 (1:1000), p-mTOR (1:1000), mTOR (1:1000), p-ULK (1:900), ULK (1:1000), p-4EBP (1:1000), 4EBP (1:1000) and Tubulin (1:1000). Afterwards, the membranes were treated with suitable HRP-linked secondary antibodies for 1 h. Protein bands were visualized using ECL chemiluminescent substrate (NCM Biotech, Cat# P10300, Suzhou, China) and quantified using ImageJ software (NIH, Bethesda, MD, USA). Detailed information on all primary and secondary antibodies is provided in Supplementary Material S1.

### 4.11. Quantitative Real-Time PCR Analysis of SASP Markers

Total RNA was extracted from HT22 cells using EZ-press RNA Purification Kit (EZBioscience, Cat# EZB-RN4, Roseville, MN, USA) following the manufacturer’s protocol. The RNA concentration and purity were determined using a NanoDrop spectrophotometer. Reverse transcription was performed using the 4× EZscript^®^ Reverse Transcription Mix II (with gDNA Remover) (EZBioscience, Cat# EZB-RT2GQ) to eliminate genomic DNA contamination and synthesize cDNA.

Quantitative real-time PCR (qPCR) was carried out using the ChamQ Universal SYBR qPCR Master Mix (Vazyme, Cat# Q711-02, Nanjing, China) on a LightCycler^®^ 96 System (Roche, Basel, Switzerland). The relative expression levels of SASP-related genes (IL-6, IL-1β, Cxcl1, TNF-α) were calculated using the 2^−ΔΔCt^ method, normalized to Gapdh. Primer sequences are listed in Supplementary Material S2.

### 4.12. Animal Model and Treatments

A total of 60 female C57BL/6 mice (SPF) (8 weeks old, 18 ± 2 g) were obtained from Guangdong Medical Laboratory Animal Centre in China. Animals were housed in a specific pathogen-free (SPF) facility under standard conditions (22 ± 2 °C, 50 ± 10% humidity, 12 h light/dark cycle) with free access to food and water. All experimental procedures involving the mice were conducted in compliance with ethical guidelines and were approved by the Institutional Animal Care and Use Committee (IACUC) at Sun Yat-sen University (Approval No. 00379609).

D-galactose (D-gal, purity ≥ 99%) was purchased from Aladdin Biochemical Technology Co., Ltd. (Shanghai, China), dissolved in sterile saline, and administered via subcutaneous injection at the nape of the neck at a dose of 1000 mg/kg/day for 6 consecutive weeks to induce an aging phenotype. α-ketoglutarate (Xintianhe, Shenzhen, China) was administered via drinking water at concentrations of 0.5% and 1% (*w*/*v*), starting concurrently with D-gal treatment. The pH value of drinking water supplemented with or without AKG was adjusted to 7.3 by the addition of sodium hydroxide.

### 4.13. Morris Water Maze (MWM)

Spatial memory in mice was evaluated using the Morris water maze (MWM) test (Nol-WM-RM, Noldus Information Technology, Beijing, China), based on previous studies [40]. The MWM test was conducted over 6 days, including 1 day of adaptation, 4 days of spatial acquisition, and 1 day of probe trial. The maze featured a pool with a diameter of 120 cm and a height of 60 cm, with the water temperature maintained at 23 ± 1 °C. To obscure the platform, non-toxic white paint was mixed into the water. Different-shaped markers were placed equidistantly around the pool walls to serve as references for the mice to locate the platform. An overhead camera was used to track and record the animals’ swimming paths automatically. Tracking software recorded each animal’s escape latency to reach the hidden platform, the time spent swimming in the target quadrant, and the number of crossings over the target platform area.

### 4.14. Rotarod Test

Motor coordination and balance were assessed using a rotarod apparatus (DXP-2, Institute of Materia Medica, Chinese Academy of Medical Sciences, Beijing, China) [41]. Mice were pre-trained for 3 consecutive days at a constant speed of 5 rpm for 300 s to ensure baseline adaptation. On the test day, mice were placed on the rotating rod, which was accelerated from 4 rpm to 40 rpm over 5 min. The latency to fall was recorded automatically for each mouse. Each animal was subjected to three trials with an inter-trial interval of at least 15 min, and the average latency was used for statistical analysis.

### 4.15. Passive Avoidance Test

The passive avoidance task was used to evaluate associative learning and memory based on aversive stimuli [42]. Mice were placed in a two-compartment shuttle box (Nol-AI-M, Noldus Information Technology, Beijing, China) consisting of a lit compartment and a dark compartment separated by a guillotine door. On the training day, each mouse was initially placed in the lit chamber. Upon entry into the dark compartment, a mild foot shock (0.3 mA, 2 s) was delivered through the grid floor. Twenty-four hours later, the mouse was reintroduced into the lit compartment, and latency to enter the dark chamber (maximum cut-off: 300 s) was recorded as a measure of memory retention. An increased latency to enter the dark compartment was interpreted as improved memory performance. All tests were conducted in a quiet environment under consistent lighting conditions.

### 4.16. Histomorphological Evaluations

For the pathological examination of the brain, samples from identical regions were excised and washed with physiological saline to eliminate residual blood and connective tissue from the surface. The samples were then fixed in 4% paraformaldehyde solution for 48 h. After routine dehydration, the tissues were embedded in paraffin and sectioned. Hematoxylin and eosin (HE) staining was applied, and the pathological morphology was examined using a light microscope.

### 4.17. Protein Extraction and DIA-Based Proteomics Analysis

Three biological samples were subjected to label-free quantitative proteomic analysis based on Data-Independent Acquisition (DIA) mass spectrometry. Briefly, samples were lysed using a lysis buffer containing 8 M urea, 1% protease inhibitor cocktail, and 50 mM Tris-HCl (pH 8.0). Protein concentration was measured using the BCA protein assay. Equal amounts of protein from each sample were reduced with dithiothreitol (DTT), alkylated with iodoacetamide (IAA), and digested with sequencing-grade trypsin at 37 °C overnight.

The resulting peptides were desalted using C18 spin columns, dried under vacuum, and resuspended in 0.1% formic acid for LC-MS/MS analysis. For DIA analysis, peptides were analyzed using a high-resolution mass spectrometer coupled with a nano-LC system. A spectral library was constructed using a pooled sample via Data-Dependent Acquisition (DDA) mode. Subsequently, all individual samples were analyzed in DIA mode using optimized variable isolation windows.

Raw DIA data were processed using the Omicsolution Platform for further analysis [43]. Protein identification and quantification were performed based on the DDA-generated spectral library. Normalization and statistical analysis were conducted on the platform to identify differentially expressed proteins between groups.

### 4.18. Statistical Analysis

The experimental data were statistically processed by biostatistical software (GraphPad Prism 9.0, San Diego, CA, USA) and expressed as mean ± SD. Unpaired Student’s test (*t*-test) was used to compare differences between two groups. The one-way analysis of variance (ANOVA) with the Bonferroni post hoc test was used to compare differences among various groups.

## Data Availability

Data is contained in the paper.

## Supplementary Materials

The following supporting information can be downloaded at https://www.mdpi.com/article/10.3390/ph18081080/s1, Supplement Material S1—Antibody. Supplement Material S2—Primer sequences.

## Abbreviations

The following abbreviations are used in this manuscript:

AKG	α-Ketoglutarate
TCA	Tricarboxylic acid
ROS	Reactive oxygen species
MMP	Mitochondrial membrane potential
SOD	Superoxide Dismutase
MDA	Malondialdehyde
PCO	Protein carbonyl
TBST	Tris-Buffered Saline with Tween^®^ 20
CCK-8	Cell Viability Assay
OCR	Oxygen Consumption Rate
SA-β-Gal	Senescence-associated β-galactosidase
D-gal	D-galactose
MWM	Morris Water Maze
HE	Hematoxylin and eosin
DIA	Data-Independent Acquisition
