# Supplementary material for: α-Ketoglutarate Attenuates Oxidative Stress-Induced Neuronal Aging via Modulation of the mTOR Pathway
Source: Pharmaceuticals (Basel). 2025 Jul 22;18(8):1080. doi: 10.3390/ph18081080 (PMC12388979; doi:10.3390/ph18081080)
Supplement: Supplementary file 1 [file pharmaceuticals-18-01080-s001.zip › Supplement material S1 -- Antibody.pdf]

	Antibody	Brand	Art.No.
primary antibodies	Rabbit Anti-phospho-eIF4EBP1 (Thr37 + Thr46) Polyclonal Antibody	Bioss	bs-3019R
	Rabbit Anti-eIF4EBP1 Polyclonal Antibody	Bioss	bs-2559R
	Rabbit Anti-phospho-ULK1 (S556)antibody	Bioss	bsm-63125R
	Rabbit Anti-ULK1 antibody	Bioss	bsm-61039R
	Phospho-mTOR (Ser2448) antibody	proteintech	80596-1-RR
	mTOR Recombinant antibody	proteintech	81670-1-RR
	p53 (1C12) Mouse mAb #2524		2524T
	GAPDH	huabio	ET1601-4-T
	α-Tubulin	Abcam	ab7291
	p21 Monoclonal antibody	proteintech	67362-1-Ig
secondary antibodies	HRP was used to label goat anti-mouse IgG	Cell Signaling Technology	7076S
	HRP was used to label goat anti-rabbit IgG	Cell Signaling Technology	7074S
