# Supplementary material for: α-Ketoglutarate Attenuates Oxidative Stress-Induced Neuronal Aging via Modulation of the mTOR Pathway
Source: Pharmaceuticals (Basel). 2025 Jul 22;18(8):1080. doi: 10.3390/ph18081080 (PMC12388979; doi:10.3390/ph18081080)
Supplement: Supplementary file 1 [file pharmaceuticals-18-01080-s001.zip › Supplement material S2 -- Primer sequences.pdf]

Table S2 Primer sequences used for qRT-PCR analysis

Gene	Forward primer (5′→3′)	Reverse primer (5′→3′)
CXCL-1	TCCGTGGCCACTGAACTG	GTGGCTATGACTTCGGTTTG
TNF-α	ATGGGCTCCCTCTCATCAGT	GCTTGGTGGTTTGCTACGAC
IL-1β	AGCTACGAATCTCCGACCAC	CGTTATCCCATGTGTCGAAGAA
IL-6	GGOCCTTGCTTTCTCTTCG	ATAATAAAGTTTTGATTATGT
GAPDH	TCATTGACCTCAACTACATGGTTT	GAAGATCGTGATGGGATTTC
